# Provider Knowledge, Attitudes, and Practices regarding Obstetric and Postsurgical Gynecologic Infections Due to Group A *Streptococcus* and Other Infectious Agents

**DOI:** 10.1155/2007/90189

**Published:** 2008-01-16

**Authors:** Chris A. Van Beneden, Lauri A. Hicks, Laura E. Riley, Jay Schulkin

**Affiliations:** ^1^Respiratory Diseases Branch, Division of Bacterial Diseases, National Center for Immunization and Respiratory Diseases, Centers for Disease Control and Prevention, Atlanta, GA 30333, USA; ^2^Department of Infectious Diseases, Warren Alpert Medical School of Brown University, Providence, RI 02903, USA; ^3^Division of Maternal-Fetal Medicine, Massachusetts General Hospital, Boston, MA 02114, USA; ^4^Department of Research, The American College of Obstetricians and Gynecologists, Washington, DC 20024, USA

## Abstract

*Background*. Knowledge, attitudes, and practices of obstetricians and gynecologists regarding the Centers for Disease Control and Prevention (CDC) recommendations for prevention of healthcare-associated group A streptococcal (GAS) infections as well as general management of pregnancy-related and postpartum infections are unknown. *Methods*. Questionnaires were sent to 1300 members of the American College of Obstetricians and Gynecologists. *Results*. Overall, 53% of providers responded. Postpartum and postsurgical infections occurred in 3% and 7% of patients, respectively. Only 14% of clinicians routinely obtain diagnostic specimens for postpartum infections; providers collecting specimens determined the microbial etiology in 28%. Microbiologic diagnoses were confirmed in 20% of postsurgical cases. Approximately 13% and 15% of postpartum and postsurgical infections for which diagnoses were confirmed were attributed to GAS, respectively. Over 70% of clinicians were unaware of CDC recommendations. *Conclusions*. Postpartum and postsurgical infections are common. Providing empiric treatment without attaining diagnostic cultures represents a missed opportunity for potential prevention of diseases such as severe GAS infections.

## 1. INTRODUCTION

Pregnant women are at risk of infection during labor and delivery; most infections of the female pelvic organs occur when normal flora of the female genital or gastrointestinal tract contaminate the normally sterile amniotic fluid and uterus. These bacteria can become pathogenic, particularly in devitalized tissue [1]. Bacterial infections in pregnant women may also originate from inoculation of bacteria during childbirth or surgery or from hematogenous spread [2]. Uterine infections are much more common after Cesarean delivery (C-section) than following a vaginal delivery [3]. Fortunately, postpartum or puerperal infections have decreased over the last hundred years due to improved procedures and effective antibiotic use. However, infections still cause about 13% of pregnancy-related deaths and are the fifth leading cause of death among this population [1].

Group A *Streptococcus* (GAS), or *Streptococcus pyogenes*, is an uncommon, but serious and potentially preventable cause of postpartum and postsurgical infections. The incidence of confirmed GAS infections among deliveries in a study in Jerusalem over 6.5 years ranged from 0.33 to 3.16 per 1000 births; suspected intra- or postpartum GAS infections occurred in 13.2% of all deliveries [4]. In a review of records of all mothers and babies with GAS bacteremia in the United Kingdom from 1980–1999, peripartum GAS infection occurred in 1 of every 11,000 live births [5]. The source of a sporadic postpartum GAS infection is typically unknown, but outbreaks of postpartum and postsurgical GAS infections have been associated with colonized healthcare workers. Healthcare workers who were asymptomatic carriers of GAS have been identified in 15 of 21 outbreaks of postpartum and postsurgical infections reported from 1976 through 2005 [6–26]; these infections represent a preventable manifestation of severe GAS disease.

In 2002, Centers for Disease Control (CDC) published guidelines for prevention of secondary invasive GAS infections, including those among postpartum and postsurgical patients [27]. Recommended strategies for the investigation of both a single case and clusters of postpartum or postsurgical GAS infection were developed. In brief, a single healthcare-associated infection should trigger notification of the hospital infection control practitioner, enhanced surveillance for additional cases and storage of GAS patient isolates for potential strain characterization. When two or more postpartum or postsurgical cases occur in the same healthcare facility within six months, CDC recommends that epidemiologic links between the cases be investigated, including screening for GAS colonization of healthcare personnel linked to known cases.

Although these guidelines were published in 2002, the level of awareness and adherence to these recommendations is unknown. Also, clinician knowledge and management of pregnancy-related and postsurgical infections has not been assessed. We conducted a survey of obstetric and gynecologic providers (OB/GYNs) to estimate the frequency of intrapartum, postpartum, and postsurgical infections they encounter to determine bacterial culturing practices among providers, and to estimate the frequency of GAS isolation among the respondents’ patient populations. We also sought to estimate awareness and adherence to the CDC guidelines, assess barriers to follow the guidelines, and query providers regarding more effective methods for distributing these recommendations.

## 2. SUBJECTS AND METHODS

In July 2005, an anonymous questionnaire focusing on current practices regarding intrapartum, postpartum, and postsurgical infections was mailed to 1300 American College of Obstetricians and Gynecologists (ACOG) Fellows and Junior Fellows in Practice; 1000 subjects were members of the Collaborative Ambulatory Research Network (CARN). Members of CARN are practicing OB/GYNs who have volunteered to participate regularly in surveys, facilitating assessment of clinical practice patterns, and aiding development of educational materials. The remaining 300 subjects consisted of a computer-generated random sample of ACOG Fellows and Junior Fellows in practice who are practicing obstetrics and/or gynecology and had not received a survey from ACOG during the previous two years (non-CARN). A second mailing was sent approximately five weeks after the first to those individuals who did not initially respond. A final reminder mailing was sent approximately six weeks later. All survey respondents who reported performing deliveries or surgeries in 2004 were eligible for inclusion in our analysis.

The survey included questions about the practitioner's age and training, his/her practice, patient population demographics, and specific questions assessing experience with diagnosing and treating intrapartum, postpartum (e.g., wound infections, endometritis, bacteremia), and postsurgical infections (e.g., wound infections, endometritis, vaginal cuff cellulitis, bacteremia), particularly infections potentially due to GAS. Survey respondents were asked to estimate the racial and ethnic distributions of their patient population, number of deliveries and surgeries performed and related infections seen during 2004. Questions also assessed the practitioner's use and understanding of the CDC guidelines for prevention of GAS infections among postpartum and postsurgical patients. Question formats were primarily multiple choice or opinion questions using a scaled response.

Survey responses were double-entered into an American Standard Code for Information Interchange (ASCII) file, corrected for data entry errors, and converted into an SAS 9 database (SAS Institute, Cary, NC, USA). The racial and ethnic characteristics of the provider's patient population were dichotomized into the following categories, based on the distribution of responses: less than 80% white versus 80% or more white; less than 25% versus 25% or more black; less than 20% versus 20% or more Hispanic; less than 7% versus 7% or more Asian/Pacific Islander. The percentage of patients with Medicaid was divided into low (<25%), medium (25–49%), and high (≥50%) categories. Differences in categorical measures were assessed using the chi-square test. The Wilcoxon 2-sample test was used to compare medians among continuous measures. All analyses were tested for significance using a P value of <.05. Comparisons of responses by CARN and non-CARN members were completed for all survey questions. All statistically significant differences between CARN and non-CARN are presented; otherwise the results for both CARN and non-CARN combined are shown.

## 3. RESULTS

### 3.1. Demographics and practice characteristics of survey respondents

Completed surveys were returned by 614 (61%) CARN and 75 (24%) non-CARN members (53% of all respondents). Respondents who had not performed deliveries or surgeries in 2004 (54 CARN and 6 non-CARN respondents) were excluded. Demographic and practice characteristics of the remaining 629 survey respondents are detailed in Table 1. A majority of respondents practiced in a group setting, worked in an urban or suburban location, and considered themselves specialists. Members of CARN were older and more likely than nonmembers to be male and to have been in practice for 10 or more years. Significant differences in sex of the respondents and number of years in practice among CARN versus non-CARN members did not persist when controlling for age: age and years of experience were highly correlated (Pearson Correlation Coefficient, R=0.91; P<.0001).

### 3.2. Obstetric and gynecologic procedures performed in 2004

Nearly all providers performed both deliveries (vaginal or Cesarean) (91%) and surgeries (hysterectomies or Cesarean deliveries) (96%) in the year preceding the survey. Based on respondents estimates, the median number (and range) of vaginal deliveries performed per provider in 2004 were 120 (4–512) (n=568 survey respondents), and a median of 55 (range: 1–450) surgeries were performed per provider in 2004 (n=596 respondents).

### 3.3. Experience with intrapartum, postpartum, and postsurgical infections

**Intrapartum infections**

Based on respondents' estimates, the median number of patients (per provider) who developed intraamniotic infection or chorioamnionitis in the year preceding the survey was five (range: 0–100). Nearly all respondents (91% of 568) stated that they would treat an intraamniotic infection empirically, without obtaining a complete blood count or blood culture or waiting for the results of either test if obtained. Over half of respondents believe that the most common bacterial cause of intraamniotic infections is polymicrobial (Table 2). With increasing years of experience, providers were more likely to consider gram-negative rods the most common bacterial cause of intraamniotic infections and less likely to consider the cause polymicrobial in origin (chi-square test for linear trend: *P* < .001 for each).

**Postpartum and postsurgical infections**

Per survey respondents, an estimated 3% of patients who had vaginal deliveries developed a postpartum infection, and 7% of patients undergoing surgery (Cesarean delivery or hysterectomy) developed postsurgical infections. Over half of providers stated that they do not obtain diagnostic specimens from their patients with postpartum infections; only 14% always obtain specimens. Providers with increasing years of experience were more likely to routinely obtain specimens for microbiological diagnosis of postpartum infections (chi-square test for linear trend: P=.002). Those who considered themselves both primary care physicians and specialists were more likely to routinely obtain cultures than respondents who considered themselves specialists only (21% versus 10%; P<.001). This association persisted when controlling for years of experience (P=.002). The suspected frequency of *Staphylococcus aureus* and group B streptococcal infections differed for postpartum compared to postsurgical infections (Table 2).

In general, more providers either always (18%) or sometimes (52%) obtained cultures from patients with postsurgical infections compared to those with postpartum infections; only 30% empirically treated without obtaining specimens. Providers with increasing years of experience were more likely to always obtain specimens from patients with postsurgical infections (chi-square test for linear trend: P=.004). As with postpartum infections, those respondents who considered themselves both primary care providers and specialists were more likely to routinely obtain specimens than those who considered themselves specialists (P=.001). When asked to estimate how many of their patients developed a postpartum infection or a postsurgical infection that was caused by GAS in the previous year, 384 and 472 providers responded, respectively. The numbers of estimated GAS-specific infections for all providers combined were 81 postpartum and 81 postsurgical GAS infections.

### 3.4. GAS screening

When GAS is isolated during routine rectovaginal culture for group B *Streptococcus* (GBS), 9% of respondents stated that they would treat the patient with antibiotics, 15% would give intrapartum antibiotic prophylaxis similar to management of GBS, most respondents (64%) would do nothing, and 8% stated that they either do not see this organism or their laboratory does not routinely test rectovaginal cultures for GAS.

### 3.5. Knowledge of GAS postpartum and postsurgical infection epidemiology

The majority of survey respondents correctly indicated that each of the five body sites listed (oropharynx, nares, rectum, vagina, and skin) may be colonized by GAS (Table 3) although less than half (47%) chose *all* five sites. Respondents chose the following as possible causes of a cluster of postpartum or postsurgical GAS infection in a facility: healthcare worker is a GAS carrier (44% respondents); patient being colonized prior to delivery or surgery (28%); as well as poor infection control practices (23%). Answers given to questions about potential sites of GAS colonization and possible causes of clusters did not vary by provider characteristics such as years in practice or practice type.

### 3.6. Adherence and barriers to following CDC's GAS guidelines

When questioned about action taken when a postpartum or postsurgical GAS infection is identified, 25% would contact infection control personnel. Few respondents chose the other two actions recommended in the CDC guidelines (save GAS isolates for further study; enhance surveillance for such infections) (Table 3). The majority (73%) of respondents stated that they had not diagnosed any GAS infections. The proportion of respondents who would take actions recommended in the guidelines increased with increasing years in practice; the proportion was also higher among providers with practices in rural areas. Few survey respondents correctly identified the number of postpartum or postsurgical GAS infections that should trigger infection control notification and investigation if the infections occurred in the same facility within six months: 23% stated correctly that two such infections should elicit this response (Table 3). CARN members (26%) more frequently chose this response than non-CARN members (4%; P<.01). No significant associations between choosing the correct response and other provider characteristics (i.e., years in practice, practice type or location) were noted.

Only 5.6% of respondents (n=611) stated that they were familiar with the 2002 CDC guidelines for management of GAS infections; in fact, over 70% were unaware of their existence. Other barriers to learning about or following the guidelines included: perceived infrequency of GAS infections in the respondent's practice (18%), publication in a journal not routinely read (13%), the fact that the provider does not consider GAS infection a priority disease (9%), and a lack of culture results for postpartum and postsurgical infections (4%). Most (74%) respondents stated that they do follow guidelines regarding diagnostic, management, or treatment of postpartum and postsurgical infections, principally those by ACOG (59%) and those which are part of hospital infection control protocols (32%).

## 4. DISCUSSION

Our study is the first published survey of the knowledge, attitudes, and practices of OB/GYNs regarding the perceived frequency, etiology, and management of intrapartum, postpartum, and postsurgical infections. Among a large number of OB/GYNs, we found that most empirically treat a variety of infections encountered in their routine practice. Although this approach is within current standards of care, this presents a missed opportunity for the identification and potential prevention of illnesses such as severe GAS infections.

Both our survey and the current literature suggest that intraamniotic, postpartum, and postsurgical infections are not uncommon. The providers in our survey identify approximately three postpartum infections per 100 vaginal deliveries and 7 postsurgical infections per 100 Cesarean deliveries and hysterectomies. These estimates are conservative, as they do not include deliveries by other providers (e.g., family practitioners), but are consistent with previous estimates in the literature. Intraamniotic infections have been reported to range from 0.5% to 10.5% of all pregnancies, occurring in 1–5% of term pregnancies and up to 10% in patients with premature labor [28–32]. Puerperal or postpartum endometritis has been reported to occur in 1–3% of women with vaginal deliveries and among 5–15% of women having scheduled repeat Cesarean deliveries [1, 28].

Nearly all survey respondents treat intrapartum, postpartum, and postsurgical infections empirically although providers are more likely to obtain diagnostic specimens from patients with postsurgical infections. While no formalized guidelines on diagnostic management of intrapartum and postpartum infections exist, empiric therapy is not contrary to current practice recommendations. Recently developed ACOG guidelines on the use of prophylactic antibiotics in labor and delivery recommend the use of prophylactic antibiotics in both high- and low-risk patients undergoing Cesarean delivery although the data to support use among low-risk patients is inconclusive [33]. Blood cultures are typically taken only when the patient does not respond to empiric antibiotics or if complications arise [34]. The rationale for not obtaining diagnostic cultures is multifold; antibiotic therapy is typically empiric and based on clinical diagnosis, patients often respond to antibiotics before culture results are known, anaerobes are notoriously difficult to isolate, accurate postpartum endometrial specimens are difficult to obtain because of possible contamination by the lower genital tract, and cultures add cost and time [35].

Conversely, pretreatment cultures facilitate management of patients who fail initial empiric antibiotic [36]. Tailoring antimicrobial therapy is impossible without identifying the etiologic agent. Investigations of the specific pathogens causing intraamniotic, postpartum, and postsurgical infections have shown that the primary causative agents change over time. For example, the principal etiology of early onset neonatal sepsis has ranged from GAS in the 1930s–1940s, *E. coli* in the 1940s–1970s, to group B streptococci in the current era [37]. Also, recent studies have documented an increase in the proportion of healthcare-associated bloodstream infections and the emergence of community-associated methicillin-resistant *Staphylococcus aureus* infections among pregnant and postsurgical patients [38–40]. Comprehensive studies to evaluate the etiologies of pregnancy-related infections have not been performed for over 15 years [41–45]. Periodic evaluations of the etiologies of pregnancy-related infections and the prevalence of antibiotic-resistant isolates among this population would be useful.

Another benefit of obtaining pretreatment cultures is that identification of certain pathogens, such as GAS, should trigger specific infection control measures to prevent spread of disease. GAS infections can be devastating with an overall case fatality ratio (CFR) of 13% and CFRs of 25–36% for the most severe manifestations—necrotizing fasciitis and streptococcal toxic shock syndrome [46]. These infections are not rare. Because we only surveyed ~3% of the nation's OB/GYNs, we estimate that as many as 2700 postpartum and 2700 postsurgical GAS infections may occur annually in the United States. Healthcare-associated transmission of GAS infections can sometimes be prevented, for example, in instances where an asymptomatic colonized healthcare worker is identified and treated as a result of a thorough epidemiologic investigation [27].

Provider responses to the GAS-specific questions included in our survey underscore both the lack of awareness of the CDC GAS guidelines among one of their targeted audiences—OB/GYNs—and potential means to correct this information gap. Very few providers were aware of the guidelines and the recommended public health action following identification of postpartum and postsurgical GAS infections. The principal reason for not understanding or following these guidelines was a lack of awareness of their existence, most likely because they were published in a journal not read by this group of providers. Potential solutions to this dilemma include publication in journals relevant to OB-GYNs, incorporation into ACOG guidelines and hospital infection control protocols, presentation at relevant meetings, and inclusion in CME lecture series.

A common thread in our survey results is the association of increasing years of experience and older age with a variety of practice patterns. Older, more experienced providers were far more likely to attempt to determine the etiology of intra- and postpartum infections and also had different beliefs from younger providers as to the specific etiologies of such infections.The reasons for these trends are unknown but may include changes in medical education or in the epidemiology of these infections.

Limitations of our survey include our low response rate, particularly among non-CARN providers although it is consistent with other ACOG surveys [47, 48]. An important limitation is that we were unable to validate the estimated number of procedures performed and actual practices reported by the respondents. It is possible that the frequencies of infections reported are significantly underestimated. Also, given the low number of providers who were aware of the CDC guidelines for postpartum and postsurgical GAS infections, attempts to estimate the respondents' understanding of specific aspects of these guidelines are of limited value.

In summary, although the use of empiric antibiotics in intraamniotic, postpartum, and postsurgical infections may currently be effective in most cases, the paucity of diagnostic cultures obtained presents a missed opportunity to monitor the etiology of these infections and to identify a potentially preventable cause of serious healthcare-associated disease—GAS infections. Current efforts to educate providers regarding the settings in which the identification of GAS should trigger a public health response and augment infection control practices are inadequate. However, our survey identified more effective means of communicating with practicing OB/GYNs and also emphasized that educational efforts should target the infection control practitioner (ICP) as most hospitals currently rely on the ICP to identify potential healthcare-associated infections and initiate necessary investigations. Periodic, time-limited studies of the etiologies of pregnancy-related infections by public health and clinical researchers would help to monitor changes in the principal pathogens, track trends in antimicrobial resistance, and guide clinical management.

## Figures and Tables

**Table 1:** Demographics and practice characteristics of survey respondents in the Collaborative Ambulatory Research Network (CARN) and non-CARN groups.

Characteristic	CARN (n=560)	Non-CARN (n=69)
Median age (in years)	48 (30–85)	40 (29–67)*
Male sex (%)	56	44^†^

Years (y) in practice:		
median (range)	16 (1–55)	6 (0–34)*
<10 y	31	57
10–19 y	32	19
≥20 y	37	25

Practice type:		
Solo or two-person practice (%)	27	25
University (%)	10	17
Multi-specialty group (%)	10	10
OB/GYN group (%)	44	39

Primary specialty:		
OB/GYN (%)	83	83
GYN only (%)	9	6
Maternal/fetal medicine (%)	5	7

Professional identity:		
Specialist (%)	63	67
Primary care & specialist (%)	35	30

Practice location:		
Urban (%)	44	42
Town, mid-sized (%)	20	23
Suburb (%)	27	26
Rural (%)	7	6

Patients receiving Medicaid (%)		
<25%	58	59
25–49%	20	17
≥50%	22	23

Race/ethnicity of patients:		
≥25% Black (%)	26	23
≥20% Hispanic (%)	27	28
≥7% Asian/Pacific Islander	19	26

* Wilcoxon 2-sample test, P<.001.

^†^ Chi-square, P=.003.

**Table 2:** Respondents' perception of most common bacterial etiology of intrapartum, postpartum, and postsurgical infections^‡^.

Most common bacterial etiology %	Type of infection		
	Intrapartum (n=572)	Postpartum (n=534)	Postsurgical (n=556)
*Staphylococcus aureus*	0.2	3	23
Group B *Streptococcus*	19	13	1
Polymicrobial	57	62	52
Gram-negative rod	22	20	19
Anaerobes	3	−^†^	−^†^
Group A *Streptococcus*	−^†^	0.8	2

* Values are %.

^†^Not applicable (not included in multiple choice).

^‡^Respondents were only allowed one choice for each type of infection.

**Table 3:** Survey respondents' knowledge and expected practice patterns regarding group A streptococcal (GAS) postpartum (PP) and postsurgical (PS) infections.

Knowledge or expected practice	CARN, %	Non-CARN, %
Which body sites can GAS colonize?* (n=569)	(n=530)	(n=66)
Oropharynx (%)	87	80
Nares (%)	88	83
Rectum (%)	75	74
Vagina (%)	78	77
Skin (%)	75	62^†^
All the above (%)	48	38

What is most likely cause of GAS PP or PS cluster in the same	(n=521)	(n=65)
facility in a 6-month period of time? (n=586)				
Poor infection control practices:	24	19
Healthcare worker is a GAS carrier:	44	42
Patient was colonized prior to delivery or surgery:	27	34
Chance:	5	6

What action do you take when you recognize a PP or PS GAS	(n=535)	(n=65)
infection among your hospitalized patients?* (n=600)				
Contact infection control:	26	15
Save isolates from patients:	9	6
Enhanced surveillance:	10	8
Give antibiotic prophylaxis to other hospitalized patients:	2	2

Take no specific action:	7	15^0§^
I have not seen any GAS infections:	74	71

What number of PP or PS GAS infections should trigger	(n=218)	(n=29)
infection control notification and investigation if occurring in				
same facility within 6 months? (n=247)		
0:	0.9	4
1:	22	17
2:	26	4^±^
3–5:	35	41
6 or more:	16	35

*Respondent could choose more than one answer.

^†^Chi-square *P* value = .03.

^§^Chi-square *P* value = .01.

^±^Chi-square *P* value < .01.
